# Potential strategies for combination therapy involving medicines with differential signal transduction regulation effects and the mechanism of action of minocycline in septic neuroinflammation

**DOI:** 10.3389/fphar.2025.1691613

**Published:** 2025-10-09

**Authors:** Kazuhiro Itoh, Hiroshi Tsutani, Yasuhiko Mitsuke, Masamichi Ikawa, Hiromichi Iwasaki

**Affiliations:** ^1^ Department of Internal Medicine, NHO Awara National Hospital, Awara, Japan; ^2^ Department of Community Health Science, Faculty of Medical Sciences, University of Fukui, Fukui, Japan

**Keywords:** brain inflammation, sepsis, minocycline, toll-like receptor 4, combination therapy

## Introduction

Sepsis-associated encephalopathy (SAE) shows diffuse dysfunctions. Hosseini et al. recently suggested that minocycline could be an effective medication for avoiding neurological complications associated with sepsis. They demonstrated that in a murine model of lipopolysaccharide (LPS) treatment, minocycline attenuated the brain inflammation and oxidative stress induced by sepsis in a dose-dependent manner (Hosseini et al., 2024). We have previously established that minocycline modulates the production of inflammatory cytokines and chemokines by inhibiting inhibitor of nuclear factor κB (IκB) kinase (IKK)α/β, a downstream signaling molecule of Toll-like receptor 4 (TLR4), in LPS-stimulated THP-1 cells (Tai et al., 2013). Based on these results, we suggest that minocycline helps the body by affecting sepsis-related brain inflammation, which is mainly controlled by microglia through TLR4-dependent pathways. This study evaluates the significance of combination therapy for SAE in two key aspects: first, the combination of minocycline with fluoroquinolones. The distinct mechanisms of action—where fluoroquinolones obstruct the upstream signaling pathway of sepsis (TLR4/MD-2 complex formation) and minocycline inhibits the downstream pathway (IKKα/β phosphorylation)—support the hypothesis that targeting various points within the same pathway amplifies the modulatory effect on neuroinflammation. Secondly, combination therapy utilizes minocycline alongside additional signal transduction pathway inhibitors. The pathophysiology of SAE encompasses the TLR4/NF-κB pathway as well as various signal transduction pathways, including mitogen-activated protein kinase (MAPK), Janus kinase/signal transduction and activator of transcription (JAK/STAT), and Nod-like receptor protein 3 (NLRP3) inflammasome, among others. Therefore, we investigated the potential of a multifaceted therapeutic strategy that integrates inhibitors aimed at each of these signaling pathways. The combination strategies outlined in this research are grounded in a theoretical framework established from current literature. This paper proposes a novel therapeutic idea, the efficacy and safety of which necessitate thorough validation through future preclinical and clinical trials.

## Pathophysiology of cytokine storms: from PAMP/DAMP recognition to inflammatory cell death

In sepsis, manifestation of a cytokine storm represents a critical and life-threatening situation. Factors that can trigger a cytokine storm include the interaction between pathogen-associated molecular patterns (PAMPs), like LPS from pathogens, and damaged cell-associated molecular patterns (DAMPs), which are produced after cells die or get hurt, along with pattern recognition receptors (PRRs). This interaction results in the overproduction of inflammatory cytokines in mammals. Cytokines are closely linked to mammalian cell death mechanisms, where cytokine signaling or PAMP/DAMP recognition can initiate inflammatory cell death. This process enhances the release of pathogenic cytokines via a positive feedback loop, ultimately resulting in the cytokine storm and causing serious damage to host tissues and organs, potentially leading to death (Karki and Kanneganti, 2021). In a mouse model of severe acute respiratory syndrome coronavirus 2 (SARS-CoV-2) infection, hemophagocytic lymphohistiocytosis, and sepsis, blocking tumor necrosis factor-alpha (TNF-α) and interferon-gamma (IFN-γ) prevents cell death and reduces the development of cytokine storm (Karki and Kanneganti, 2021). These data suggest that inhibiting the overproduction of inflammatory cytokines is crucial to improving the prognosis of sepsis.

## Mechanism of action of minocycline and fluoroquinolones through the inhibition of the TLR4/NF-κB pathway

Monocytes, macrophages, and microglia express PRRs such as TLR4, which identify PAMPs and DAMPs during sepsis, subsequently generating inflammatory cytokines and chemokines via intracellular signaling pathways. In SAE, microglia serve a crucial role in neuroinflammation. The role of TLR4 in neuroinflammation is context-dependent and not uniform. A study has indicated that TLR4 activation may confer neuroprotective effects under specific circumstances (Radpour et al., 2022). In Gram-negative bacterial sepsis, increased TLR4 activation induced by LPS, a principal component of Gram-negative bacteria, is regarded as a key contributor driving the detrimental neuroinflammatory cascade seen in SAE.

In an LPS sepsis model utilizing THP-1 cells as a monocyte surrogate, we demonstrated that minocycline dose-dependently inhibited the production of inflammatory cytokines such as TNF-α, interleukin (IL)-6, and IFN-γ, as well as chemokines including IL-8 and interferon-inducible protein (IP)-10 (Tai et al., 2013). The immunomodulatory effects of minocycline extend beyond the previously reported LPS-stimulated THP-1 cell model and the LPS sepsis mouse model described by Hosseini et al. The neuroprotective effects and anti-inflammatory properties have been investigated for decades across many disease models. Likewise, the immunomodulatory effects of fluoroquinolones have been reported. To elucidate the context of the discussion in this study, we summarize the pertinent research findings below (Table 1). According to Table 1, the neuroprotective effects of minocycline are multifaceted, indicating that its mechanism of action is not restricted to the inhibition of the TLR4 pathway by LPS stimulation but also involves the TLR2 pathway in response to stimulation by various pathogens (*Staphylococcus aureus*) (Zou et al., 2025). Moreover, it has been established that the activation of microglia can be inhibited in non-infectious conditions, such as cerebral ischemia (Yrjänheikki et al., 1998). The consistent findings indicating that minocycline mitigates neuroinflammation across multiple models support its potential as a treatment candidate for SAE. This study concentrates on the TLR4 pathway, crucial in Gram-negative bacterial sepsis associated with LPS. Fluoroquinolones represent a theoretically advantageous upstream inhibitor for minocycline. We suggest an innovative treatment theory utilizing fluoroquinolones that inhibit upstream TLR4 signaling by binding to the TLR4 co-receptor, MD-2, thereby preventing the formation of the LPS-TLR4 complex (Zusso et al., 2019). This unique approach, focusing on the first phase of the signaling cascade, complements minocycline’s effect on the downstream pathway (IKKα/β phosphorylation) (Tai et al., 2013). This dual-target strategy constitutes the fundamental justification for our proposed combination.

**TABLE 1:** Key studies on the role of minocycline and fluoroquinolones in neuroinflammation.

Authors (year)	Study type	Experimental model	Key findings	Relevance to this study
Minocycline				
Hosseini et al. (2024)	*In vivo*	Mouse LPS sepsis model	Minocycline decreased brain inflammation and oxidative stress in a dose-dependent fashion and facilitated recovery in mice	This offers concrete data supporting the hypothesis of this study, specifically that minocycline inhibits septic neuroinflammation
Tai et al. (2013)	*In vitro*	Human monocyte-like cell line (THP-1 cells)	Minocycline inhibited the synthesis of inflammatory cytokines triggered by LPS stimulation. This effect was facilitated by the suppression of IKKα/β phosphorylation downstream of TLR4	This provides direct evidence for the mechanism suggested in this study, specifically that minocycline inhibits downstream signaling of the TLR4/NF-κB pathway
Zou et al. (2025)	*In vivo*/*in vitro*	Mouse *Staphylococcus aureus* infection model/microglia cells	Minocycline demonstrated neuroprotective benefits through the modulation of the TLR2 and STAT3 pathways	This article is crucial for illustrating the variety of mechanisms of action and broadening the discussion to pathways beyond TLR4
Yrjänheikki et al. (1998)	*In vivo*	Rat cerebral ischemia model	Minocycline inhibited microglial activation and caspase-1 expression following cerebral ischemia, exhibiting neuroprotective effects	A notable piece of evidence indicates that minocycline has been recognized for its role as an inhibitor of microglial activation for an extended period
Fluoroquinolone				
Zusso et al. (2019)	*In vitro*	Primary cultured microglia	Ciprofloxacin and related drugs interact with the TLR4 co-receptor MD-2, blocking the assembly of the LPS-TLR4 complex and thereby reducing NF-κB activation and cytokine synthesis	This presents significant evidence supporting the combination therapy concept that fluoroquinolones impede the upstream TLR4/NF-κB pathway

## Antibacterial efficacy of the combination use of tetracycline and fluoroquinolone

Minocycline and fluoroquinolones exhibit considerable overlap in their antimicrobial spectra, and our literature review failed to find any definitive evidence concerning their synergistic antimicrobial activity; however, we found several studies of efficacy in specific situations (Supplementary Table S1). It is important to acknowledge that these clinical instances do not directly pertain to SAE. Although they present intriguing instances illustrating the possible advantages of combining tetracycline and fluoroquinolone drugs, their pathophysiology circumstances markedly diverge from SAE. Therefore, caution must be observed when generalizing these findings to the management of SAE.

*Stenotrophomonas maltophilia* exhibits significant susceptibility to trimethoprim-sulfamethoxazole (TMP/SMX), the primary therapy. The IDSA guidelines recommend the combination of the following medications for *S. maltophilia*: TMP/SMX, minocycline, levofloxacin, cefiderocol, or the combination of ceftazidime/avibactam and aztreonam (Tamma et al., 2024).

In the context of brucellosis treatment, patients receiving the doxycycline-rifampin combination therapy had a significantly higher recurrence rate than those receiving doxycycline-rifampin-levofloxacin combination therapy (22.6% vs. 9.3%, p-value = 0.01). The incidence rate of treatment-related side effects did not exhibit a statistically significant difference (20.4% vs. 11.3%, p-value = 0.059) (Hasanain et al., 2016).

In comparison to levofloxacin monotherapy, the combination of minocycline and levofloxacin markedly postponed the development of levofloxacin-resistant strains in clinical isolates of *Elizabethkingia anophelis* and inhibited the establishment of DNA gyrase mutations even after numerous passages (Lee et al., 2024).

A randomized controlled trial of *Helicobacter pylori* eradication therapy demonstrated that a four-drug regimen incorporating tetracycline and levofloxacin as a salvage therapy for primary treatment failure significantly enhanced eradication rates compared to the standard four-drug regimen of clarithromycin and amoxicillin (82.1% vs. 70.1%, P = 0.0001) (Alavinejad et al., 2023).

Japanese spotted fever (JSF), a rickettsial illness, exemplifies sepsis by inducing an excessive release of inflammatory cytokines and chemokines during disease onset (Tai et al., 2014). In a recent meta-analysis of body temperature data from JSF case reports, we identified a significant hypothermic effect in the tetracycline and fluoroquinolone combination group in comparison to the tetracycline monotherapy group. This serves as a specific example of clinical cases receiving the combination of tetracycline and fluoroquinolones (Itoh et al., 2023).

Although there is no established evidence that the combination of tetracycline and fluoroquinolones augments antibacterial efficacy concerning SAE, it is intriguing to observe that the benefits of combining these two antibiotics have been demonstrated in several clinical cases.

## A framework for combination therapy targeting interconnected signaling networks in SAE

**FIGURE 1:**
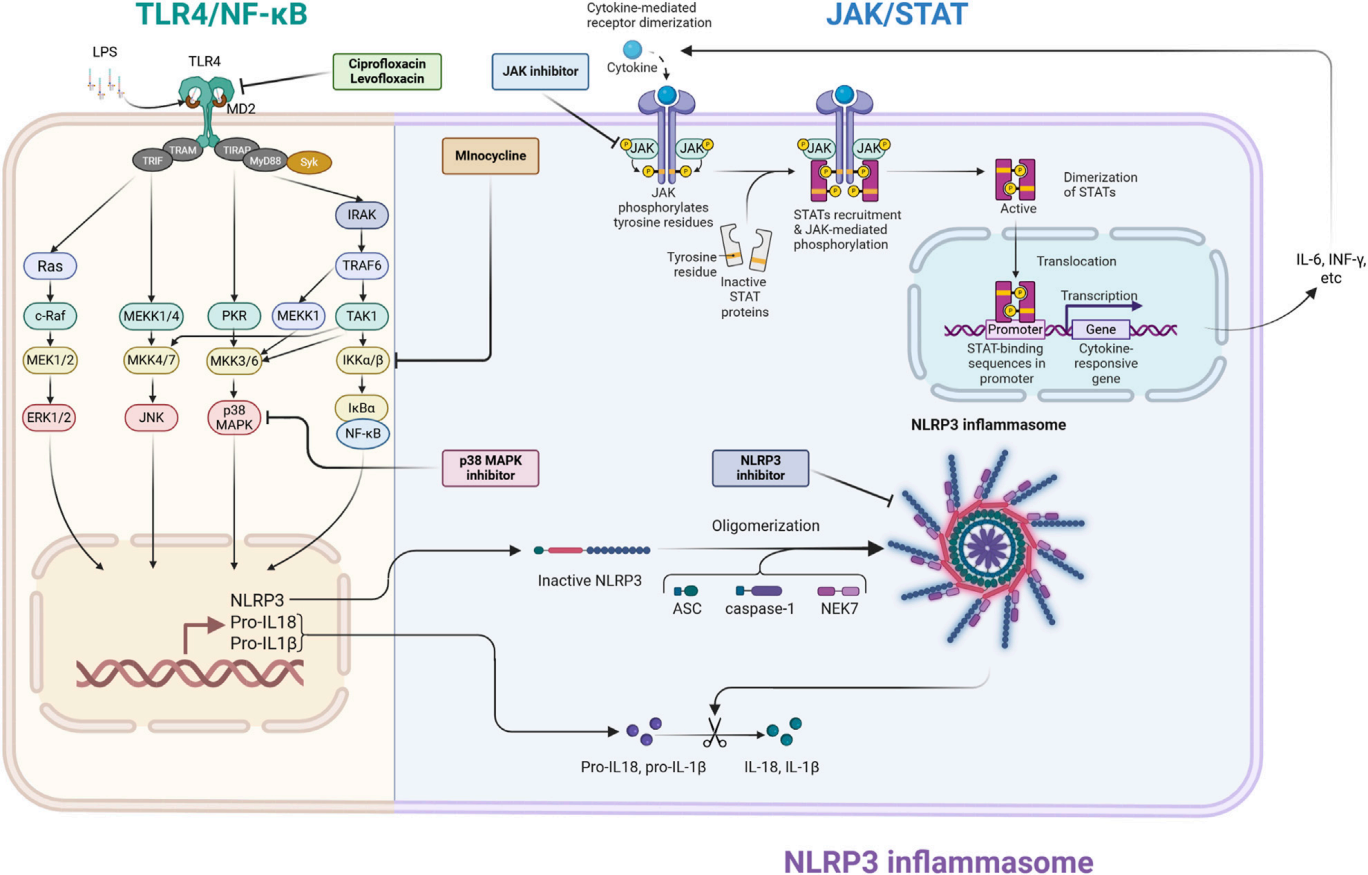
Schematic illustration of principal neuroinflammatory signaling pathways and treatment targets in septic encephalopathy (SAE). This illustration depicts the interactions among the three principal inflammatory signaling pathways (TLR4/NF-κB, NLRP3 inflammasome, and JAK/STAT) implicated in the pathogenesis of SAE, along with the pharmacological targets for these pathways. TLR4/NF-κB signaling pathway: Bacterial-derived lipopolysaccharides (LPS) interact with the TLR4/MD2 complex, initiating intracellular signaling pathways that result in the nuclear translocation of the transcription factor NF-κB. This facilitates the transcription of inflammatory cytokines and the precursors of NLRP3 inflammasome substrates, pro-IL-1β and pro-IL-18 (priming signal). Fluoroquinolone drugs (ciprofloxacin, levofloxacin) obstruct the interaction of LPS with TLR4 at the proximal segment of this route. This pathway also stimulates the MAPK pathway, which is inhibited by p38 MAPK inhibitors. NLRP3 inflammasome: NLRP3 assembles an inflammasome complex with ASC and caspase-1 in response to diverse intracellular stressors. Activated caspase-1 cleaves pro-IL-1β and pro-IL-18, resulting in the production and release of the potent inflammatory cytokines IL-1β and IL-18 (activation signal). NLRP3 inhibitors impede the assembly of this complex. JAK/STAT signaling pathway: Inflammatory cytokines (IL-6, IFN-γ, etc.) generated by NF-κB attach to cell surface receptors, activating JAKs linked to these receptors, which subsequently phosphorylate STATs. Activated STATs dimerize and translocate to the nucleus, facilitating the synthesis of supplementary inflammatory cytokines. This operates as a positive feedback loop that significantly enhances the inflammatory response. JAK inhibitors are proposed to obstruct JAK activation, whereas minocycline may impede receptor activation by cytokines. This illustration visually represents the theoretical foundation for “multimodal combination therapy,” which integrates pharmacological agents with distinct modes of action to tackle the intricate pathophysiology of SAE. ASC, apoptosis-associated speck-like protein containing caspase recruitment domain; ERK, extracellular signal-regulated kinase; IFN-γ, interferon-gamma; IκBα, inhibitor of nuclear factor kappa-B kinase alpha; IKKα/β, IκB kinase alpha/beta; IL-6, interleukin-6; IRAK, IL-1 receptor-related kinase; JAK, Janus kinase; JNK, c-Jun N-terminal kinase; MAPK, mitogen-activated protein kinase; MEKK, mitogen-activated protein/ERK kinase kinase; MD2, myeloid differentiation factor 2; MKK, mitogen-activated protein kinase kinase; MyD88, myeloid differentiation factor 88; NEK7, NIMA-related protein kinase 7; NF-κB, nuclear factor kappa-B; NLRP3, Nod-like receptor protein 3; PKR, double-stranded RNA-activated protein kinase; STAT, signal transduction and activator of transcription; Syk, spleen tyrosine kinase; TAK1, transforming growth factor-β-activated kinase 1; TIR, Toll/interleukin-1 receptor; TIRAP, TIR domain-containing adaptor protein; TLR, toll-like receptor; TRAF, tumor necrosis factor receptor associated factor; TRAM, TRIF-related adaptor molecule; TRIF, TIR domain-containing adaptor protein inducing interferon-β. The figure was created using BioRender (https://biorender.com/).

The TLR4/NF-κB pathway is a vital starter in the pathogenesis of SAE, operating within a complex, interrelated signaling network. To enhance treatment efficacy, it is essential to acknowledge the broader network that includes the major MAPK, JAK/STAT, and NLRP3 inflammasome pathways (Chen et al., 2020; Ren et al., 2020). Figure 1 depicts the principal signaling pathways related to SAE.

These pathways establish a harmful feedback loop. Activation of TLR4 can initiate the MAPK pathway, which enhances cytokine production. These cytokines subsequently stimulate the JAK/STAT pathway, functioning as a potent “amplification loop” for the inflammatory response. Concurrently, danger signals arising from cellular damage can trigger the NLRP3 inflammasome, resulting in the generation of powerful cytokines such as IL-1β and IL-18. The suppression of each of these pathways has exhibited neuroprotective effects in preclinical SAE models (Liu et al., 2022; Bhadauriya et al., 2025; Chen et al., 2020; Zhong et al., 2020).

Given the significant interplay among various systems, a multifaceted therapeutic strategy is a more logical approach than focusing on a singular channel. We offer a strategy for combination therapy that concurrently targets both the “initiation signals” and the “amplification signals” of neuroinflammation. For instance, the combination of minocycline (a modulator of the TLR4 pathway) with a JAK inhibitor could simultaneously reduce the initial inflammatory stimulus and the next cytokine-mediated amplification loop. This method may provide substantial neuroprotective effects that monotherapy cannot provide. This idea necessitates validation in forthcoming trials to ascertain the efficacy and safety of these combination treatments in pertinent SAE models.

## Adverse events (particularly in antibiotics)

A cohort study revealed that the median age (IQR) of 2,780 SAE patients was 67 (56–76.8) (Lu et al., 2023). Another study into pediatric SAE revealed that children exhibit heightened vulnerability to metabolic irregularities typically observed in PICUs, thereby elevating their risk of developing SAE relative to adults. The limited incidence of reported cases and fatalities from pediatric SAE is largely attributable to underreporting or underdiagnosis, underscoring this as a significant clinical concern (Dumbuya et al., 2023). Furthermore, numerous studies have expressed apprehensions over adverse events linked to the use of antibiotics in pediatric SAE patients (Chen et al., 2022; de Araújo et al., 2022; Gong et al., 2022; Dumbuya et al., 2023).

Minocycline may result in tooth discoloration, enamel hypoplasia, and temporary bone growth abnormalities in children, particularly those under 8 years of age during dental development, as indicated in the manufacturer’s package insert. Moreover, fluoroquinolones may impact joints and cartilage throughout pediatric development, and their administration in children is strictly prohibited save for specific exceptions (e.g., tosufloxacin). Furthermore, concerns regarding adverse events in geriatric patients, including tendinopathy and central nervous system effects, are extensively reported and restrict their application in another significant SAE demographic. The substantial safety issues, especially in at-risk pediatric and geriatric groups, provide a considerable obstacle to the clinical implementation of this suggested combination therapy and must be carefully evaluated against any potential advantages. As a result, meticulous selection and use of antibiotics are crucial for pediatric patients.

Secondly, the risk of antibiotic resistance arising from microbiome perturbations is a universal concern for all antimicrobial drugs, and the significance of cautious antimicrobial usage is widely acknowledged. Specifically, concurrent use of two or more antibacterial agents increases the likelihood of adverse effects. This encompasses significant disruptions of the gut microbiota, potentially resulting in problems such as *Clostridioides difficile* infection, and may have enduring effects on host immunity. Moreover, the synergistic pressure of two antibiotics might expedite the emergence and selection of multidrug-resistant organisms, a significant public health issue. Therefore, the unnecessary application of such combination antimicrobial therapy must be stringently avoided and should only be contemplated when a definitive, significant advantage is expected.

## Limitations

This document aims to suggest a novel treatment hypothesis grounded in existing knowledge, rather than to present new experimental results. Minocycline and fluoroquinolones may have synergistic effects in protecting against sepsis-associated neuroinflammation through distinct mechanisms. However, there is presently no direct experimental evidence, so this suggestion remains a working hypothesis at present. This remains a hypothesis that necessitates further validation via such studies as animal experiments using the SAE model mice and clinical trials involving humans. Tetracycline and fluoroquinolones target distinct components of the TLR4 pathway; nonetheless, SAE entails intricate interactions with additional signaling pathways. Thus, it is essential to evaluate solutions that address not only the impacts on the TLR4 pathway but also more extensive modes of action. The pathophysiological mechanisms of JSF and other bacterial situations, and SAE are distinct, thereby restricting the relevance of the data to SAE.

## Conclusion

We suggest a mechanism via which minocycline exerts an inhibitory effect on neuroinflammation in SAE. This study primarily identified the inhibitory mechanism as depending on the TLR4 signaling pathway. Ultimately, this paper presents a hypothesis-generating proposal that is intended to promote additional research. Future research is expected to examine therapeutic possibilities that involve the manipulation of various signaling pathways associated with SAE and to substantiate the notions outlined above.
