# Supplementary material for: Potential strategies for combination therapy involving medicines with differential signal transduction regulation effects and the mechanism of action of minocycline in septic neuroinflammation
Source: Front Pharmacol. 2025 Oct 9;16:1691613. doi: 10.3389/fphar.2025.1691613 (PMC12546914; doi:10.3389/fphar.2025.1691613)
Supplement: Supplementary file 1 [file Table1.docx]

**Supplemental Table S1. Clinical evidence for monotherapy versus combination therapy with tetracycline and fluoroquinolone.**

| **Agents ( monotherapy/combination therapy)** | **Target disease/microorganism** | **Clinical background** | **Summary of results** | **References** |
| --- | --- | --- | --- | --- |
| Combination therapy (minocycline, levofloxacin) | *Stenotrophomonas maltophilia* | Nosocomial infection (resistant Gram-negative bacilli) | The IDSA guidelines recommend minocycline and levofloxacin as alternate therapies. | Tamma PD, et al. (2024) |
| Combination therapy (levofloxacin + minocycline) vs monotherapy (levofloxacin) | *Elizabethkingia anophelis* | Inhibition of levofloxacin resistance | Combination therapy markedly delayed the development of resistant bacteria and inhibited the acquisition of DNA gyrase mutations in contrast to levofloxacin monotherapy. | Lee CC, et al. (2024) |
| Combination therapy (tetracycline + levofloxacin) vs standard therapy | *Helicobacter pylori* | Secondary decontamination in instances where the primary decontamination has failed (salvage therapy) | The combination therapy of tetracycline and levofloxacin demonstrated a significantly superior eradication rate compared to the conventional four-drug regimen (82.1% vs. 70.1%). | Alavinejad P, et al. (2023) |
| Combination therapy (doxycycline-rifampicin-levofloxacin) vs dual therapy (doxycycline-rifampicin) | Brucellosis | Treatment of acute/subacute brucellosis | The three-drug combination therapy group exhibited a significantly reduced recurrence rate post-treatment in comparison to the two-drug combination therapy group (9.3% vs. 22.6%). | Hasanain A, et al. (2016) |
| Combination therapy (tetracycline + fluoroquinolone) vs monotherapy (tetracycline) | Japanese spotted fever (Rickettsial infection) | infectious disease presenting with sepsis-like symptoms | A meta-analysis of case reports indicated that combination therapy had significantly greater antipyretic benefits compared to monotherapy. | Itoh K, et al. (2023) |
